# Rate of reimbursement for 22-modifier in shoulder surgery

**DOI:** 10.1016/j.xrrt.2024.12.007

**Published:** 2025-01-24

**Authors:** Walter R. Smith, Allyson N. Pfeil, Matthew A. Coker, Pito Huerta, Davin K. Fertitta, Corey F. Hryc, T. Bradley Edwards, Michael C. Cusick

**Affiliations:** a Fondren Orthopedic Research Institute, Fondren Orthopedic Group, Texas Orthopedic Hospital, Houston, TX, USA; b Texas A&M School of Engineering Medicine, Houston, TX, USA; c University of Nevada, Reno School of Medicine, Reno, NV, USA

**Keywords:** 22-modifier, Total shoulder arthroplasty, Orthopedic reimbursement, Complexity modifiers, Medicare reimbursement rates, Compensation

## Abstract

**Background:**

The 22-modifier is a reimbursement amendment designed by the Current Procedural Terminology (CPT) to reflect increased case complexity. When a CPT code is shared between more than 1 procedure or is used to capture a breadth of procedures, a 22-modifier can be used to acknowledge the increased workload in a particular procedure when compared to the standard procedure. We hypothesize that discrepancies exist among 22-modifier reimbursement rates in shoulder surgery, and that payers, particularly commercial, are reimbursing at lower rates for extensive surgical efforts. Identifying potential reimbursement shortcomings can open dialog between payers and surgeons to ensure transparency and fairness.

**Methods:**

22-modifier amendments for total shoulder arthroplasty (TSA) (CPT code 23472), revision of TSA (23474), and arthroscopic rotator cuff repair (29827) occurring from October 31, 2018 to March 23, 2022 were queried, resulting in 566 instances from 11 surgeons at a single site. Financial data were collected from the billing department, while patient demographics and operative reports were collected from medical records. The billing staff requested reimbursement identically on all claims, excluding 1 surgeon, who also sent a reimbursement cover sheet detailing case complexity. Request for reimbursement was submitted for some cases without an operative report. Complexity justifications included obesity (body mass index >30 or >35), reverse TSA, revision procedures, massive repair, surgeon-determined prolonged length of procedure, no justification for 22-modifier listed, and undiagnosed hypertension which created a medical emergency.

**Results:**

In total, 150 (26.5%) of 22-modifier cases were successfully reimbursed. TSA, revision of TSA, and arthroscopic rotator cuff repair had a reimbursement rate of 40.7%, 35.3%, and 13.0%, respectively. Of successful claims, Medicare reimbursed 75.3% and commercial only 26.7%. The highest rates of reimbursement justifications were length of procedure (41.7%), reverse shoulder arthroplasty (40.6%), and revision procedure (32.4%). The surgeon who included the cover sheet was successfully reimbursed (41.6%) more frequently than 2 surgeons with similar case volume (18.3% and 19.5%).

**Conclusion:**

Criteria for successful reimbursement of the 22-modifier are ambiguous, complicating reimbursement efforts. Clinicians should consider concentrating efforts on obtaining 22-modifier reimbursement from Medicare in cases with increased length of procedure, as well as revision procedures and reverse shoulder arthroplasties. Surgeons may receive higher reimbursement rates with the addition of a cover sheet detailing the complexity of the procedure and any associated increases in complication rates or costs. Clarification from insurance carriers is needed to determine what constitutes a 22-modifier.

Despite an increasing prevalence of inpatient and outpatient orthopedics, medical reimbursement rate by payers is declining.^3^^,^^5^^,^^6^^,^^12^^,^^15^^,^^17^^,^^20^^,^^24^ Even cost-efficient outpatient orthopedic clinics are experiencing declining reimbursements.^23^ Thus, addressing fair and adequate physician compensation, particularly related to coding, is increasingly critical. In addition to these reimbursement woes, heterogeneity exists among shoulder procedures by Current Procedural Terminology (CPT) codes. For instance, shoulder arthroplasty performed in the setting of fracture reconstruction may involve longer lengths of procedures and require more effort than cases for osteoarthritis.^25^^,^^26^ However, these procedures are both included under the same CPT code. Additionally, equivalently coded anatomic total shoulder arthroplasty (aTSA) and reverse shoulder arthroplasty (RSA) procedures involve different indications, techniques, and outcomes.^7^^,^^14^^,^^16^^,^^29^ This surgical variety complicates the reimbursement process for surgeons further.

To address the ongoing debate over payment reduction and the unique characteristics of specific cases, modifiers such as 22 have been incorporated into the CPT to designate case complexity.^1^ The American Association of Professional Coders (AAPC) published literature outlining appropriate use, instructing physicians to document (1) why the case would be considered complex, (2) surgeon intervention, and (3) additional length of procedure requirements.^8^ Physicians typically provide this justification in the operative note.^13^ The AAPC recognized 3 correct instances of the 22-modifier: (1) increased intensity or length of procedure, (2) increased difficulty or effort, and (3) severe patient condition.^8^ However, despite the seemingly broad applicability of these criteria to many cases, the Centers for Medicare and Medicaid Services (CMS) reports that reimbursement will only be granted under “very unusual circumstances”.^21^

Of submitted 22-modifiers, a modest 27%-42% are approved for additional reimbursement, and many researchers conclude seeking this additional reimbursement may not be worth the additional administrative labor.^4^^,^^11^^,^^18^^,^^27^^,^^30^^,^^31^ Conversely, a study that positively encouraged surgeons to seek reimbursement reported morbid obesity as most probable for reimbursement in hip arthroplasty.^30^ Neither the AAPC nor CMS explicitly cite obesity as an appropriate justification for 22-modifier^8^^,^^21^ despite the fact that obesity may adversely impact surgical outcomes.^2^^,^^15^^,^^28^ On the contrary, other researchers have found anatomical variations, such as abnormal bony or soft-tissue degradation, are the most likely reason to be reimbursed.^11^^,^^27^

The discrepancy in the current literature regarding appropriate 22-modifier justifications is foundational for our study, in which we assess 22-modifier reimbursement rates among the most commonly performed shoulder surgeries. Congruent with prior literature, we anticipate that there will be differences among 22-modifier reimbursement by justifications and payer types.^11^^,^^27^^,^^30^ In general, negotiated Medicare rates have been steadily declining, and these rates are often significantly lower than those of commercial plans.^9^^,^^12^^,^^17^^,^^22^^,^^25^ Taking this into account, we hypothesize that commercial plans will monetarily reimburse less, and Medicare will demonstrate the highest 22-modifier reimbursement success rate. Analyzing and reporting these 22-modifier reimbursement rates may provide physicians with a better understanding of reimbursement criteria and where to focus efforts for increased reimbursements.

## Methods

Following institutional review board approval, an internal database was queried for 22-modifier amendments on shoulder surgeries from October 31, 2018 to March 23, 2022 by 11 surgeons at a single orthopedic specialty hospital. Shoulder surgeries were identified by CPT code: 23472 (primary or reverse TSA), 23474 (revision TSA), and 29827 (arthroscopic rotator cuff repair). Next, medical charts were reviewed for demographic information (age, sex, and body mass index [BMI]), as well as the operative report which provided the justification for 22-modifier. Finally, the clinic’s financial department provided claim details, including insurance carrier, and request status.

In total, 567 records using the 22-modifier were initially identified, and one record was excluded for being from a self-pay patient. Thus, 566 records were analyzed. Eleven operative notes were not found; these operative notes were in a separate hospital system that the finance department (those who submitted the reimbursement request) could not access. Thus, 11 claims using the 22-modifier were submitted without including an operative report. The finance department billed every claim the same, except for records from Surgeon 7, who also provided a detailed cover sheet for reimbursement submission (Fig. S1). This cover sheet illustrated the differences between aTSA and RSA in terms of surgical difficulties, extent of pathology, and outcomes.

The operative notes were scanned for 22-modifier justifications and classified as follows: obesity, use of reverse prosthesis (RSA), revision procedure, massive repair, increased procedure length, no justification, and undiagnosed hypertension. Obesity was surgeon-defined as a threshold of either ≥30 or ≥35 BMI meeting or exceeding the World Health Organization designation that involved difficulties in exposure, repair, and case management. Use of a reverse prosthesis corresponded with RSA implantation, reporting operative and outcome differences compared to aTSA. Revision was defined as any explicit mention of revision repair or arthroplasty that increased complexity and risk due to scarring, retained hardware, or altered anatomy. Repair of a massive rotator cuff was subjective to the surgeon, generally defined as either an exceptionally sizeable or numerous tears that increased the technicality, length of procedure, and method. Tear classification was diagnosed intraoperatively but visually estimated to be exceeding 5 cm. Length of procedure was defined as the surgeon reporting of increased operative duration without explicit mention of the procedure type (revision, RSA, or otherwise). No justification was defined as an operative report with no supplementary information on case complexity and no mention of 22-modifier. Undiagnosed hypertension was a unique cohort in which the surgeon described intraoperative difficulties in managing the case considering this unexpected condition. Operative note excerpts were identified and, when possible, the same surgeon, payer type, and justification were presented for comparison.

Data were analyzed using a combination of Microsoft Excel (Microsoft Corp., Redmond, WA, USA), Python v3.11 (Python Software Foundation, Beaverton, OR, USA), and GraphPad (GraphPad Software, Boston, MA, USA). Statistical tests, including *t*-tests, logistic regression, and Chi-square were assessed for significance at *P* < .05. Confidence intervals were calculated at a 95% confidence level.

## Results

### Payout overview

The 566 records were comprised of 221 arthroplasties (39.0%) (208 RSAs and 13 aTSAs), 68 revision arthroplasties (12.0%), and 277 arthroscopic rotator cuff repairs (48.9%). In total, 150 of 566 (26.50%) 22-modifier cases were reimbursed. Primary TSA (23472) contained the highest reimbursement success rate (40.72%), followed by revision TSA (23474) and rotator cuff repair (29827) at 35.29% and 13.00%, respectively (Table I). Of TSA, the reimbursement rates for anatomical and reverse were 23.1% and 41.8%, respectively. Successful reimbursements were associated with older age (70.19 vs. 62.91, *P* < .0001), lighter BMI (28.95 vs. 30.91, *P* < .005), and a high ratio of female (58.0% vs. 45.7%, *P* < .01) patient records, confirmed by logistic regression (*P* < .01) (Tables II and III). Primary TSA was found to be 3.45x more likely to be reimbursed (*P* < .05), with no other differences in CPT codes found. The operative notes of successfully reimbursed records had fewer words than the unsuccessfully reimbursed (*P* < .05, 1104.12 vs. 1208.48, respectively). Surgeon payout rate varied; the 3 highest volume surgeons had rates of 41.57%, 18.32%, and 19.54%, respectively (Table IV).

**Table I:** Successful 22-modifier reimbursements by procedure type.

Procedure	CPT code	Reimbursement rate (%)
Total shoulder arthroplasty	23472	90 (40.72)
Revision total shoulder arthroplasty	23474	24 (35.29)
Arthroscopic rotator cuff repair	29827	36 (13.00)

*CPT*, Current Procedural Terminology.

**Table II:** Demographic overview of 22-modifier patients by 22-modifier success rate.

	Successful reimbursement (n = 150)	Unsuccessful reimbursement (n = 416)	*P* value
Age	70.19 ± 8.90	62.91 ± 9.73	**<.0001**
BMI	28.95 ± 5.99 (n = 144)	30.91 ± 6.53 (n = 404)	**.0017**
Male:female ratio	63:87	226:190	**.0096**
Word count of operative note	1104.12 ± 407.44 (n = 147)	1208.48 ± 432.88 (n = 408)	**.0112**

*BMI*, body mass index.

Bold *P* values represent statistical significance.

**Table III:** Logistic regression analysis of statistically significant patient characteristics.

	Odds ratio [CI]	*P* value
Age	1.0909 [1.07-1.12]	**<.0001**
BMI	0.9501 [0.92-0.98]	**.0013**
Sex∗	1.6426 [1.13-2.40]	**.0095**

*BMI*, body mass index; *CI*, confidence interval.

Bold *P* values represent statistical significance.

∗ Male sex for reference.

**Table IV:** 22-modifier success rate by surgeon.

Doctor ID (n)	Successful reimbursements (%)
Surgeon 1 (33)	4 (12.12)
Surgeon 2 (35)	3 (8.57)
Surgeon 3 (17)	3 (17.65)
Surgeon 4 (1)	0 (0)
Surgeon 5 (1)	0 (0)
Surgeon 6 (40)	9 (22.50)
Surgeon 7 (178)	74 (41.57)
Surgeon 8 (131)	24 (18.32)
Surgeon 9 (87)	17 (19.54)
Surgeon 10 (38)	16 (42.11)
Surgeon 11 (5)	0 (0)

*ID*, identification.

### Payer overview

Of 150 reimbursed cases, 113 were paid by Medicare and 37 by commercial payers (Table V). The success rate for all Medicare claims was 42.64% compared to 13.91% for all commercial claims, where logistic regression confirmed Medicare was 3.31x more likely to reimburse than commercial (*P* < .05). Neither Medicaid nor Worker’s compensation (11 and 24 cases, respectively) reimbursed any cases. Overall, commercial records were of younger age (58.89 vs. 72.09, *P* < .0001) than Medicare, with no significant differences of age, sex, or BMI between reimbursed and unreimbursed commercial claims (Table VI, Supplementary Table S1). Of Medicare claims, records of older age were statistically significant to be reimbursed more (73.74 vs. 70.86, *P* < .001) as well as lower BMI records (28.62 vs. 30.66, *P* < .05).

**Table V:** Payer analysis by 22-modifier success rate.

Payer type (n)	Successful claims (%)
Commercial (266)	37 (13.91)
Medicaid (11)	0 (0)
Medicare (265)	113 (42.64)
Workers’ compensation (24)	0 (0)

**Table VI:** Patient demographics (age, sex, and BMI) categorized by insurance type.

Characteristic	Commercial	Medicare	Medicaid	Worker’s compensation
Age	58.89 ± 7.79	72.09 ± 6.70	55.64 ± 11.65	54.88 ± 6.37
M:F ratio	162:104	105:160	6:5	16:8
BMI	30.51 ± 5.98	29.80 ± 6.61	30.78 ± 6.84	35.50 ± 7.31

*BMI*, body mass index; *M*, male; *F*, female.

### Justification overview

**Figure 1:**
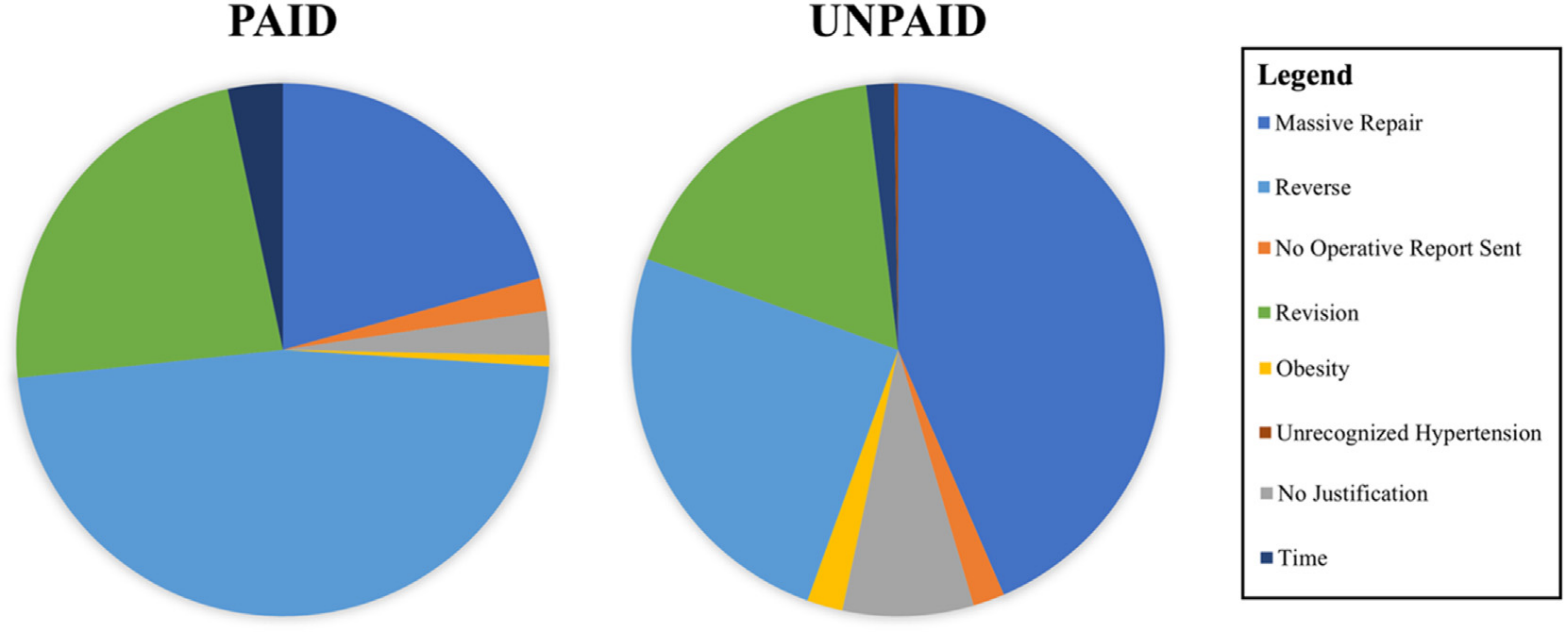
Count of paid and unpaid claims by justification.

Reimbursement rates by justifications were as follows: length of procedure (41.67%), reverse prosthesis (40.57%), revision (32.41%), no operative report sent (27.27%), massive repair (14.62%), no justification (10.81%), obesity (10%), and undiagnosed hypertension (0%) (Table VII, Fig. 1). Payer analysis revealed commercial plans reimbursed for massive repair, no justification, reverse prosthesis, and revision, while Medicare reimbursed all justifications except undiagnosed hypertension (Table VII). Logistic regression revealed no statistical differences of justification type and reimbursement outcome.

**Table VII:** Rates of reimbursement of 22-modifier by justifications.

Justifications (n)	Overall reimbursement rate (%)	Commercial (%)	Medicare (%)
Massive repair (212)	31 (14.62)	16 (51.13)	15 (48.39)
No operative report sent (11)	3 (27.27)	0 (0.00)	3 (100.00)
No justification (37)	4 (10.81)	2 (50.00)	2 (50.00)
Obesity (10)	1 (10.00)	0 (0.00)	1 (100.00)
Reverse prosthesis (175)	71 (40.57)	9 (12.68)	62 (87.32)
Revision (108)	35 (32.41)	10 (28.57)	25 (71.43)
Time (12)	5 (41.67)	0 (0.00)	5 (100.00)
Unrecognized hypertension (1)	0 (0.00)		

Reimbursed cases are then broken down by payer type (commercial and Medicare).

## Discussion

This study found that the 22-modifier reimbursement rate of common shoulder surgeries was only 26.50%, with procedure length, use of reverse prosthesis, and revision procedure justifications having the highest frequency of reimbursement. We expected Medicare to have the highest reimbursement rate and found 75.33% of all successful claims were reimbursed by Medicare with 42.64% of all Medicare claims reimbursed. Included patient records were nearly 65 years old on average; thus, a significant portion of the study population would be expected to be on Medicare. The practice is a high-volume, orthopedic surgery specialty hospital, with an average patient age of 64.5 ± 13.3 years in 2023, where 51% of payers are commercial, 44.7% Medicare, 2.6% Medicaid, and <2% Worker’s compensation. Regarding justification, reimbursement was most successful for length of procedure, reverse prosthesis, and revision, possibly indicating payer’s understanding of physician workload. Records of cases with additional reimbursement approved had a lower BMI and were older in age. These BMI findings are likely secondary to the patient’s age, as older patients experience more muscle wasting and are generally lighter.

### Payer type

Medicare and commercial plans were the only payers to reimburse for 22-modifier. However, the payout rate was starkly different between the 2 (42.64% vs. 13.91%, respectively). Furthermore, a similar study in joint replacement reported a Medicare reimbursement rate of 69.6%.^11^ This amount of variation signals a disconnection between payers and providers regarding the complexity modifier.

Medicare reimbursed not only more often but also more broadly in justifications than commercial payers. Commercial payers reimbursed more frequently for massive repairs; however, every other justification reimbursement rate was led by Medicare. Since commercial plans constitute a higher deficit and surplus than Medicare, perhaps these payers are less willing to reimburse due to paying higher rates overall.^19^^,^^22^

### Justifications

Procedure length, reverse prosthesis, and revision were the 3 most successfully reimbursed justifications. Overall, massive repairs were reimbursed strikingly low when considering the total number of requests. One potential explanation includes the lack of explicit size criteria for tears mentioned in the justification request. Payer clarification would likely increase these massive repair reimbursement rates. For example, a massive tear has been previously defined as a tear >5 cm in anterior to posterior diameter, a complete tear involving 2 or more tendons, or a nonacute tear retracted to the glenoid.^10^ Although the surgeons visually used this assessment, mention of approximate size or tendon involvement may provide additional, necessary details to payers.

The finding that successfully reimbursed operative reports contained fewer words was surprising. Initially, one may suspect payers value succinct writing. However, justification excerpts are quite similar between successful and unsuccessful reimbursements. One physician wrote, verbatim, the same justification paragraph for 2 cases billed to the same payer but was only reimbursed for one (Supplementary Table S2). One excerpt justifying a massive repair provided more detail into the injury and the surgical intervention yet failed to get reimbursed while a relatively shorter and simpler explanation was successfully reimbursed. Thus, there likely exist alternate explanations for reimbursement rates than simply the justification in the operative report.

In the case of Surgeon 7’s unusually high rates and volume, Surgeon 7 submitted an additional cover sheet that further emphasized case complexity, surgical technique, and postoperative case management strategies. Considering that this is the only difference between Surgeon 7 and the rest of the surgeons, it is likely that this cover sheet had an impact on reimbursement success. Surgeons may consider implementing this technique to increase the chance of a successful 22-modifier reimbursement.

Another potential factor regarding 22-modifier reimbursement success may lie in the way Medicare and other insurers classify patients. Medicare defines Hierarchal Condition Categories of medical conditions and then derives a Risk Adjustment Factor of the patient.^32^ This allows CMS to plan presumably increased services for more ill patients than healthy. Although used primarily for costs and budgeting, CMS could consider the Risk Adjustment Factor of the patient when determining the appropriateness of 22-modifier. Most commercial payers do not factor in comorbidities with their reimbursement structure.

### Comparison with prior studies

Our finding of an overall additional reimbursement rate of 26.5% is similar to other specialties^11^^,^^18^^,^^27^ that report rates at 42%, 31%, and 27% in spine/total joint replacement, urology, and total joint arthroplasty, respectively. Such variation in reimbursement rates is indicative of opaque guidelines and low reimbursements for complexity. Differences in rates of additional reimbursement may be a consequence of time. Richman et al^27^ reported a 42% reimbursement rate from 2004 to 2011, while Lotan et al^18^ reported 31% from 2006 to 2007. Interestingly, the rate of 27% from Comrie et al^11^ is from the same time period as this study (2018-2022), which may indicate that 22-modifier reimbursement rates have been declining like other rates.

### Applications of findings

Payers should be more transparent regarding complex reimbursements and what constitutes use of a 22-modifier. Publishing more specific guidelines for appropriate use and justification of 22-modifier requests could streamline efficiency for both the payer and the surgeon. The results of this study suggest additional reimbursement is not expected in younger patients with commercial insurance plans. Based on our findings, surgeons should consider concentrating reimbursement attempts with Medicare and commercial plans for justifications involving increased length of procedure, revision procedures, and use of a reverse prosthesis. In the absence of specific guidelines, surgeons should also proactively consider the use of a cover sheet (Fig. S1) to potentially increase the chance of successful reimbursement. Based on our data and other literature,^11^ it is not recommended to request reimbursement from worker’s compensation or Medicaid. Unfortunately, failure to be compensated for cases with increased complexity may disincentivize clinicians from treating this patient population.

The present study represents the 22-modifier reimbursement rates at a single orthopedic specialty hospital. During the period of the study, the institute handled all 22-modifier reimbursements in the financial department. Thus, the findings will be most applicable to institutions with similar billing procedures and volume. Of note, subsequent to data collection and analysis for this study, our institution has recently contracted with a third-party billing service that manages 22-modifier reimbursements and has achieved a much higher rate of reimbursement than those reported here.

### Limitations

This study has several limitations. First, all cases occurred at a single site, in a major metropolitan area in the United States, which may affect the generalizability. Each surgeon used their own criteria for determining the appropriateness of the 22-modifier; as such, all cases were analyzed as one cohort reflecting a total population of the institute to mitigate any case discrepancies. Justifications, particularly obesity and massive repair, were determined by the individual surgeons and did not explicitly mention the size of the patient or the tear. Therefore, different criteria for complexity among surgeons may have over-represented or under-represented the justification breakdowns. No separate analysis between Medicare Advantage and standard Medicare was conducted. No denial or appeal information was obtained or analyzed. Finally, justifications were assessed using researcher discretion; some operative reports listed several justifications as to the appropriateness of the 22-modifier. In these circumstances, the justification that appeared to have been the overarching theme was selected as encompassing for the case. Despite these limitations, the group is confident in the accuracy and validity of the results reported.

## Conclusion

The 22-modifier was found to have a modest 26.50% payout for various shoulder arthroplasties and procedures. Older, lighter, and female patients were reimbursed disproportionately higher, as well as records billed to Medicare. Increased length of procedure, use of reverse shoulder prosthesis, and revision procedure justifications had the highest reimbursement rate. Finally, a detailed cover sheet on the procedure appeared to increase reimbursement rates. The authors advocate for payer systems to publish the specific criteria used to determine whether to reimburse for a 22-modifier to help guide surgeons in appropriate usage.

## Acknowledgment

The group acknowledges the surgical effort by the 11 physicians which led to this study. Surgeries that led to this research project were performed at the Fondren Orthopedic Group, a division of Ortho Lone Star, which shares a location with Texas Orthopedic Hospital, an HCA Hospital.

## Disclaimers:

Funding: The Fondren Orthopedic Research Institute at the Texas Orthopedic Hospital supported a portion of the study team.

Conflicts of interest: The senior author is a paid consultant for DJO/Enovis, Medacta, and Aevumed, but this did not impact the study. The other authors, their immediate families, and any research foundation with which they are affiliated have not received any financial payments or other benefits from any commercial entity related to the subject of this article.
