# Supplementary material for: Rate of reimbursement for 22-modifier in shoulder surgery
Source: JSES Rev Rep Tech. 2025 Jan 24;5(2):186–91. doi: 10.1016/j.xrrt.2024.12.007 (PMC12047554; doi:10.1016/j.xrrt.2024.12.007)
Supplement: Supplementary Figure S1 [file mmc1.docx]

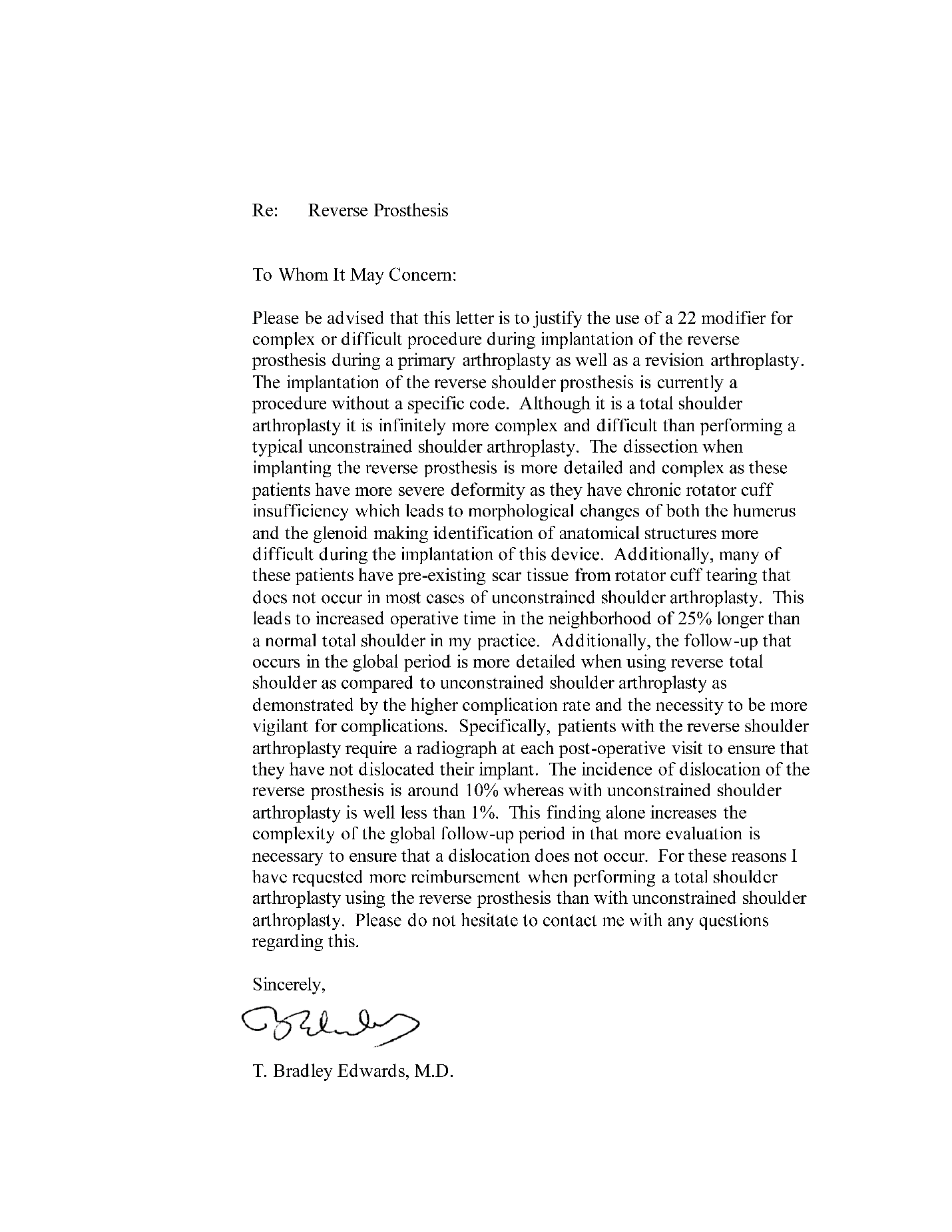


**Supplementary Figure 1.** Cover Sheet Submitted by Surgeon 7 to Enhance 22-Modifier Reimbursement Request for Reverse Total Shoulder Arthroplasty.
