# Supplementary material for: Rate of reimbursement for 22-modifier in shoulder surgery
Source: JSES Rev Rep Tech. 2025 Jan 24;5(2):186–91. doi: 10.1016/j.xrrt.2024.12.007 (PMC12047554; doi:10.1016/j.xrrt.2024.12.007)
Supplement: Supplementary Table S1 [file mmc2.docx]

**Supplementary Table 1.** Comparison of patient demographics (age, sex, and BMI) based on insurance type and reimbursement outcome. Bold P values indicate statistical significance <.05.

| Characteristic | Commercial Paid (n=37) | Commercial Unpaid (n=229) | P Value | Medicare Paid (n=113) | Medicare Unpaid (n=152) | P Value |
| --- | --- | --- | --- | --- | --- | --- |
| Age | 59.32 ± 6.17 | 58.82 ± 8.03 | .7179 | 73.74 ± 6.43 | 70.86 ± 6.65 | **<.001** |
| M:F Ratio | 22:15 | 140:89 | .8463 | 41:72 | 64:88 | .3379 |
| BMI | 29.98 ± 4.69 | 30.59 ± 6.17 | .5659 | 28.62 ± 6.33 | 30.66 ± 6.70 | **.0127** |
