# Supplementary material for: Rate of reimbursement for 22-modifier in shoulder surgery
Source: JSES Rev Rep Tech. 2025 Jan 24;5(2):186–91. doi: 10.1016/j.xrrt.2024.12.007 (PMC12047554; doi:10.1016/j.xrrt.2024.12.007)
Supplement: Supplementary Table S2 [file mmc3.docx]

**Supplement Table 2.** Excerpts from Operative Reports by Surgery, Physician, and Justification.

| Case Details | Excerpt |
| --- | --- |
| Surgeon 7  Payer: Medicare  Claim Status: Paid  CPT: 23472  Justification: Reverse | The reverse prosthesis is a much more complex device than conventional shoulder arthroplasty requiring more time for insertion as well as more diligent follow up to observe for the higher complication rate that can occur in the postoperative global period. For these reasons, I feel the use of a 22 modifier is indicated when coding for this procedure. |
| Surgeon 7  Payer: Medicare  Claim Status: Denied  CPT: 23472  Justification: Reverse | The reverse prosthesis is a much more complex device than conventional shoulder arthroplasty requiring more time for insertion as well as more diligent follow up to observe for the higher complication rate that can occur in the postoperative goal period. For these reasons, I feel the use of 22 modifier is indicated when coding for this procedure. |
| Surgeon 8  Payer: Medicare  Claim Status: Paid  CPT: 29827  Justification: Massive Repair | Please note modifier 22 will be appended to the 29827 because of the complexity and difficulty with extra work required to repair both the subscapularis and supraspinatus, which required different approaches and repair techniques. |
| Surgeon 8  Payer: Medicare  Claim Status: Denied  CPT: 29827  Justification: Massive Repair | Modifier 22 used because of extensive adhesions and had to be released and multiple passes of the suture and complex technique was required because of the delaminated tear. Several passes had to be done more than once in order to pass through the delaminated structures and identifying the anatomy was very difficult due to the duration between injury and surgery. Approximately 25% to 30% more time was required for this procedure than standard rotator cuff repair. |
| Surgeon 10  Payer: Medicare  Claim Status: Paid  CPT: 29827  Justification: Obesity | Required more than 50% typical time for rotator cuff repair given morbid obesity, massive retracted tears and the need for a complex double row repair along with seven arthroscopic anchors including the subscapularis repair. |
| Surgeon 10  Payer: Medicare  Claim Status: Denied  CPT: 23472  Justification: Obesity | A 22 modifier was indicated for this increased complexity. Preoperative assessment, intraoperative complications, postoperative complication profile for this complex reverse shoulder arthroplasty in a patient with morbid obesity and wheelchair dependence. |
| Surgeon 6  Payer: Commercial  Claim Status: Paid  CPT: 23472  Justification: Revision | We elected for a small shell and the small patient's anatomy, morcellized bone graft was impacted into the proximal humerus, all the previous arthroscopic anchors and sutures were carefully removed. This involved excess time due to the revision nature of the surgery and qualify for a 22 modifier in this procedure. |
| Surgeon 6  Payer: Commercial  Claim Status: Denied  CPT: 23474  Justification: Revision | Due to the extensive revision nature of this procedure, this took additional time greater than 45 minutes, necessitating a 22 modifier for this case. |
| Surgeon 6  Payer: Medicare  Claim Status: Paid  CPT: 23474  Justification: Time | This extensive dissection and scar and adhesion removal took an excess amount of time and added greater than 1 hour to the procedure, which qualifies for this procedure for a 22 modifier. |
| Surgeon 6  Payer: Medicare  Claim Status: Denied  CPT: 23474  Justification: Time | This involves an hour of operative time when it was extremely complex to extract the stem. This extensive scar removal as well as removal of well fixed cemented stem requires time and complexity that justifies a 22 modifier. |
